# Delayed Expanding Intracerebral Pneumatocele Following Severe Craniofacial Trauma: A Case Report With Serial Imaging Correlation

**DOI:** 10.7759/cureus.108709

**Published:** 2026-05-12

**Authors:** Sneha N Nadar, Umashankar Baskar, Venkata Sai

**Affiliations:** 1 Radiodiagnosis, Sri Ramachandra Institute of Higher Education and Research, Chennai, IND

**Keywords:** delayed complication, delayed pneumocephalus, intracerebral pneumatocele, pneumocephalus, serial ct imaging, skull base fracture, tension pneumocephalus

## Abstract

Pneumocephalus is a common complication of craniofacial trauma; however, delayed progressive enlargement resulting in intracerebral pneumatocele represents an uncommon and potentially life-threatening condition.

We report a case of a 27-year-old male with severe traumatic brain injury following a road traffic accident with extensive skull base fractures. Initial imaging demonstrated hemorrhagic contusions with small intracranial air foci that resolved on early follow-up. Subsequently, delayed reappearance and progressive enlargement of air in the basifrontal region, manifesting as an intracerebral pneumatocele and likely related to persistent communication with the frontal sinus, resulted in significant mass effect with midline shift and subfalcine herniation. Surgical repair of the anterior cranial fossa defect was performed, resulting in clinical and radiological improvement.

Delayed expanding intracerebral pneumatocele is a critical complication of skull base trauma. Serial imaging and close clinical monitoring are essential for early detection and timely management.

## Introduction

Pneumocephalus is defined as the presence of intracranial air and is a well-recognized entity most commonly encountered following craniofacial trauma, neurosurgical procedures, or infections involving the paranasal sinuses and skull base [1]. It is particularly associated with fractures of the anterior cranial fossa and paranasal sinuses, which create a direct communication between the intracranial compartment and extracranial air space [2]. In most cases, pneumocephalus is small, asymptomatic, and resolves spontaneously without intervention [3,4]. However, in certain situations, progressive accumulation of intracranial air may occur, leading to tension pneumocephalus, a potentially life-threatening condition characterized by mass effect, raised intracranial pressure, and neurological deterioration [5].

The pathophysiology of pneumocephalus has been explained by two principal mechanisms: the ball-valve mechanism, wherein air enters the cranial cavity through a dural defect but is unable to escape, and the inverted bottle mechanism, in which cerebrospinal fluid (CSF) leakage creates negative intracranial pressure, resulting in passive entry of air [6]. These mechanisms are especially relevant in patients with complex skull base fractures, persistent dural defects, or ongoing CSF leaks.

Intracerebral pneumatocele is a rare and less frequently described variant, characterized by localized accumulation of air in the intracranial cavity within the brain parenchyma that may behave as a space-occupying lesion [7]. Unlike simple pneumocephalus, these lesions can progressively enlarge over time due to continued air entry and impaired resorption, potentially resulting in significant mass effect and even brain herniation if left untreated [8]. Delayed presentation of intracerebral pneumatocele is uncommon and may pose a diagnostic challenge, particularly when it occurs after apparent initial clinical and radiological improvement [9].

This case highlights the delayed evolution of post-traumatic pneumocephalus into an expanding intracerebral pneumatocele following apparent initial radiological resolution of pneumocephalus, emphasizing the importance of serial imaging surveillance and early recognition of persistent skull base communication in patients with evolving neurological symptoms.

## Case presentation

A 27-year-old male presented following a road traffic accident involving a two-wheeler collision, sustaining injuries to the head, face, and chest, associated with a history of loss of consciousness and altered sensorium for two hours. There were no known significant comorbidities.

On clinical examination, swelling and abrasion were noted over the right forehead and temporal region. Neurological assessment revealed severe traumatic brain injury with a Glasgow Coma Scale (GCS) score of E1VTM4 at presentation. The patient was intubated and placed on ventilatory support due to a compromised airway and altered mental status in the setting of polytrauma.

Initial CT imaging (day 0): April 2025

**Figure 1:**
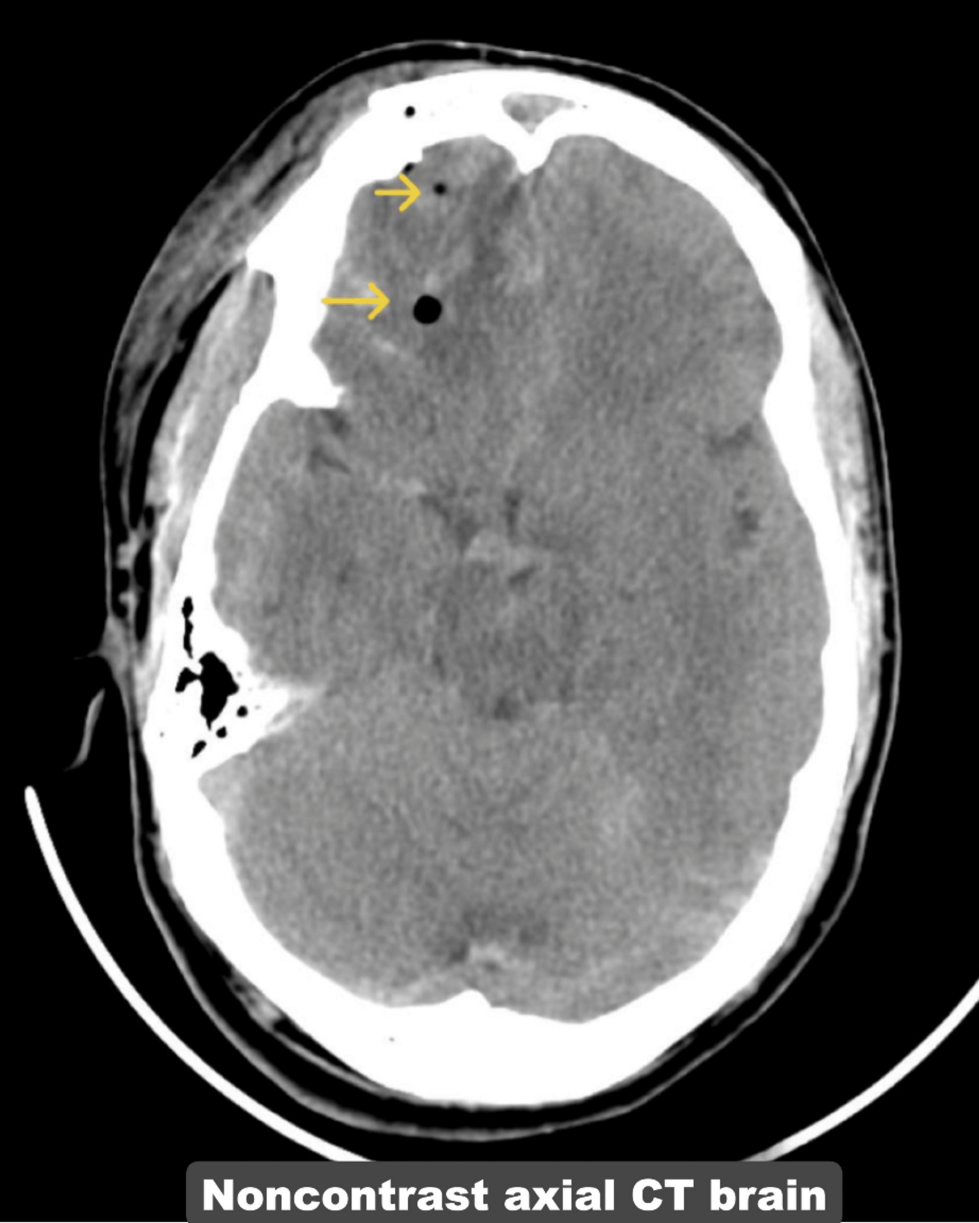
Day 0 initial non-contrast axial CT brain showing frontal contusions with air foci Yellow arrows show intracranial air foci (pneumocephalus) with surrounding hemorrhagic contusions in the right frontal region.

At presentation, non-contrast computed tomography (CT) of the brain revealed acute hemorrhagic contusions involving the right frontal and basifrontal lobes with associated intracranial air foci/pneumocephalus (Figure 1). Additional findings included subarachnoid, extradural, and subdural hemorrhages, along with a midline shift of approximately 4 mm toward the left side. Extensive comminuted skull base fractures involving the paranasal sinuses were noted, with associated hemosinus.

Early follow-up (day 0, 10 hours): April 2025

On early follow-up, the patient remained hemodynamically stable with no evidence of new neurological deterioration (GCS E2VTM4). Ventilatory support was continued, and there was no significant change in clinical status compared with initial presentation.

**Figure 2:**
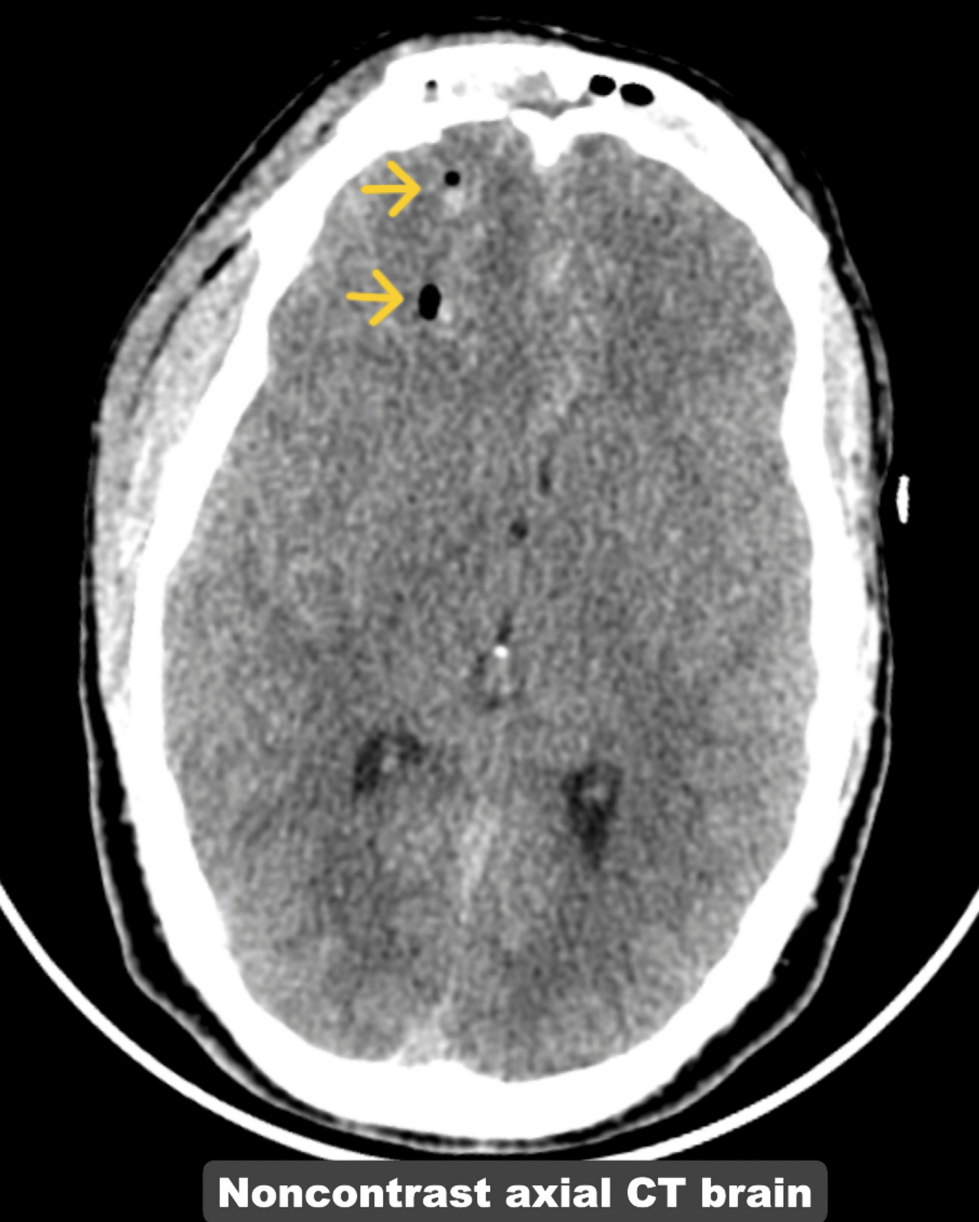
Day 0 (10 hours) non-contrast axial CT brain showing persistence of intracranial air foci Yellow arrows show persistent intracranial air foci (pneumocephalus) with a mild increase in the surrounding hemorrhagic contusions in the right frontal region.

Repeat non-contrast CT of the brain demonstrated a mild increase in the size of the hemorrhagic contusions and extradural hemorrhage, with persistence of previously noted intracranial air foci (Figure 2).

Day 2 follow-up: April 2025

On Day 2, the patient showed mild improvement in sensorium with a stable neurological status (GCS E2V2M5-6). There were no new clinical concerns, and the overall condition appeared to be improving compared with prior assessments.

**Figure 3:**
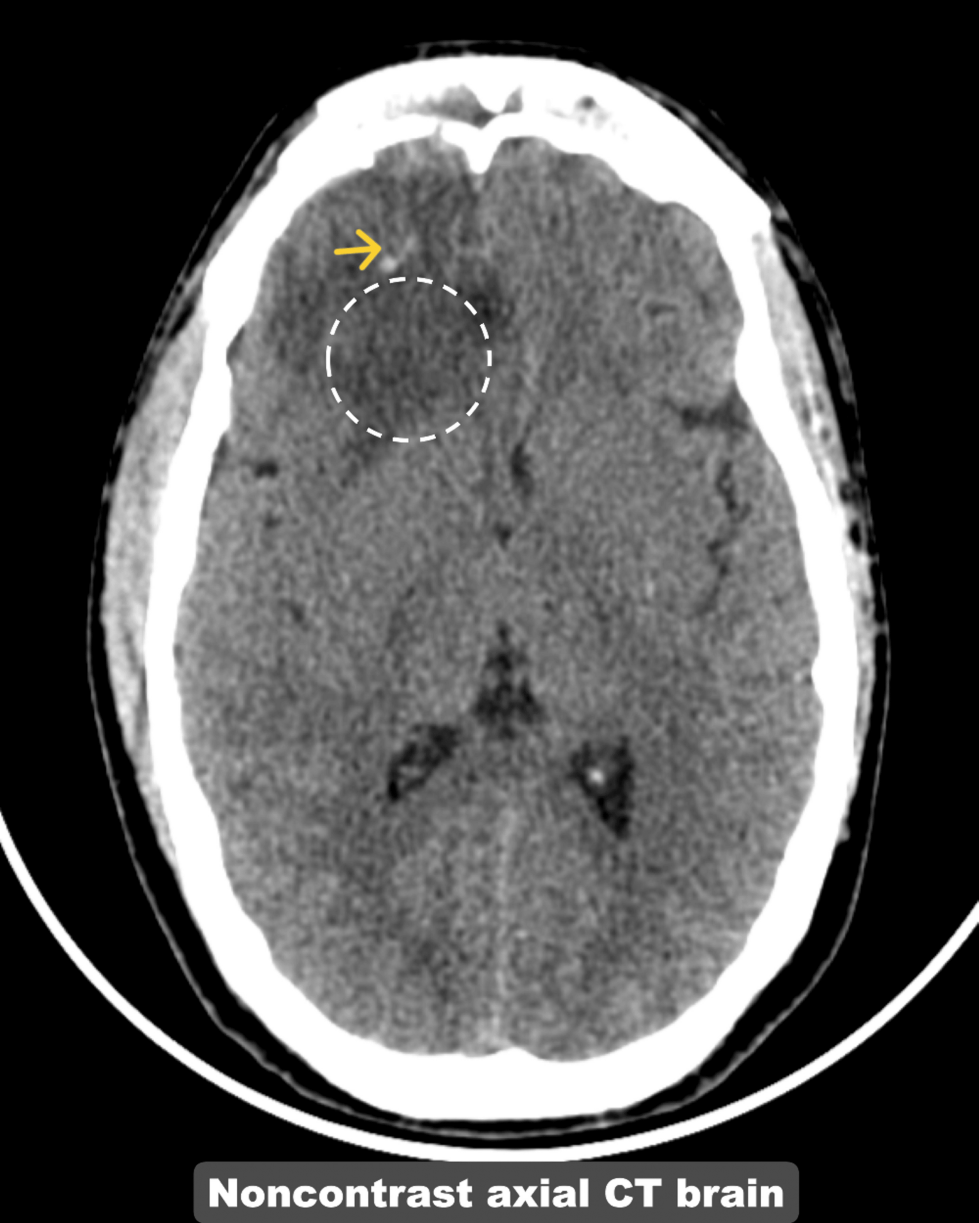
Day 2 non-contrast axial CT brain showing resolution of initial pneumocephalus Yellow arrow shows interval reduction in the previously noted hemorrhagic contusion in the right frontal region. White dotted circle shows complete resolution of the earlier identified intracranial air foci (pneumocephalus).

**Figure 4:**
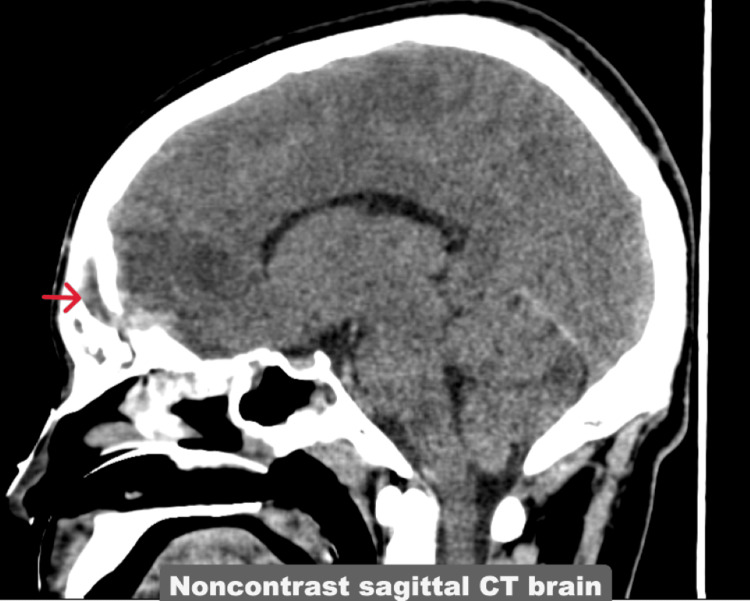
Day 2 non-contrast axial CT brain showing persistent right frontal sinus hemorrhage Red arrow shows persistent right frontal sinus hemorrhage.

Repeat non-contrast CT of the brain demonstrated interval reduction in the previously noted hemorrhagic components, along with complete resolution of the earlier identified intracranial air foci (Figure 3). However, persistent right hemosinus is noted (Figure 4).

Day 14 follow-up: May 2025

On Day 14, the patient developed a low-grade fever with fluctuating sensorium (GCS E3V3M5) for one day. No other significant clinical examination findings were observed.

**Figure 5:**
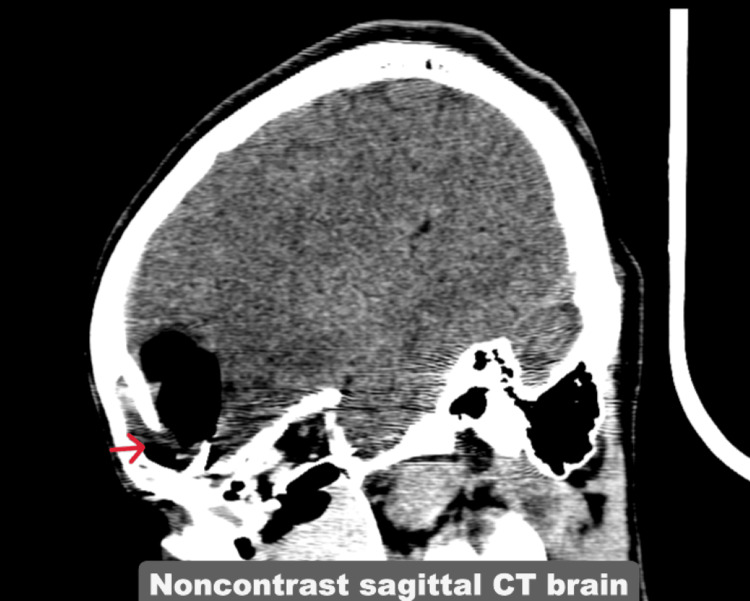
Day 14 non-contrast sagittal CT brain image demonstrating partial resolution of the right frontal hemosinus Red arrow shows partial resolution of the right frontal hemosinus, unmasking a communication pathway for intracranial air entry and resulting in formation of a small intracranial pneumatocele.

**Figure 6:**
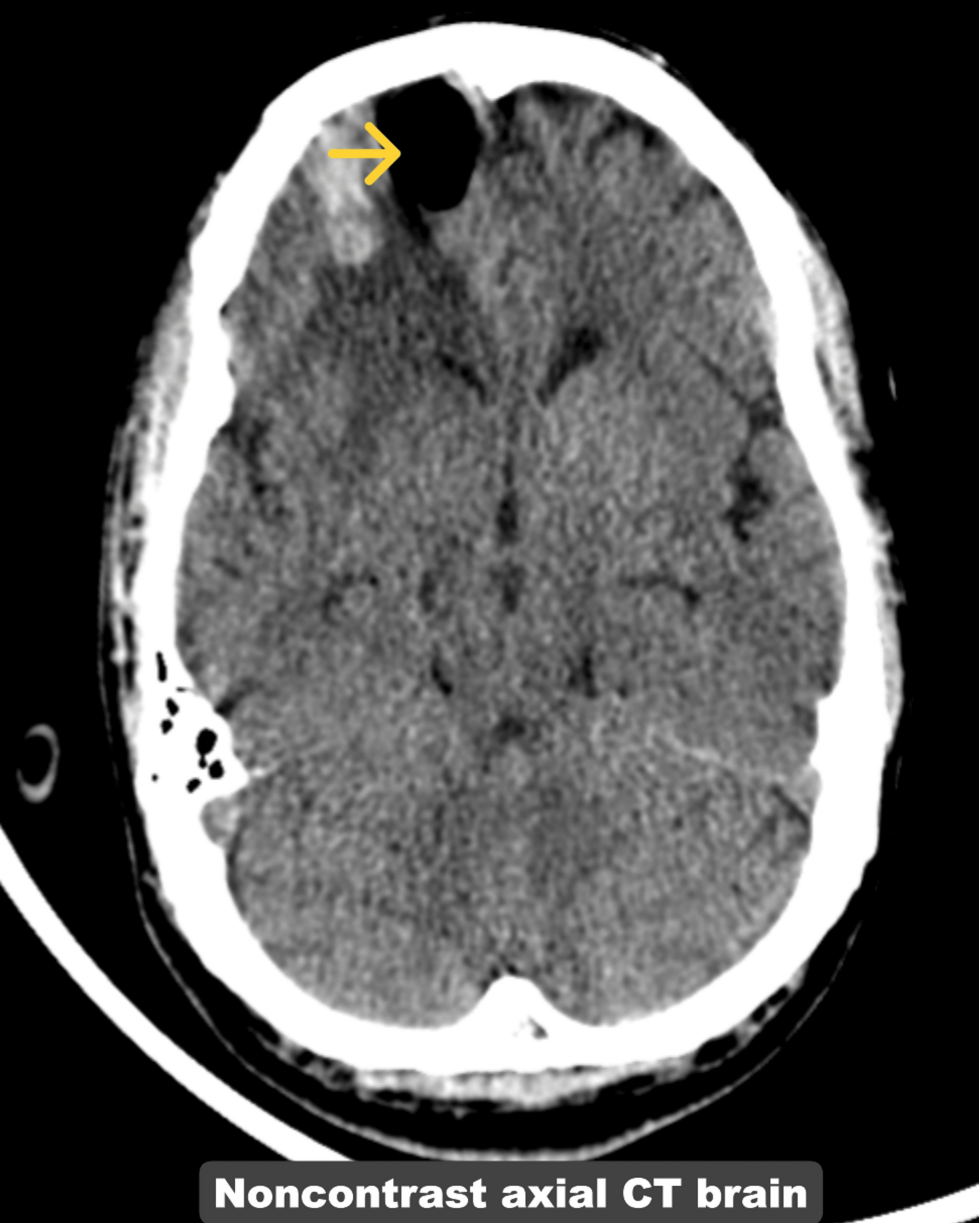
Day 14 non-contrast axial CT brain showing new basifrontal air collection (intracerebral pneumatocele) The yellow arrow shows a new air collection (intracerebral pneumatocele) in the right basifrontal region.

Repeat non-contrast CT of the brain demonstrated partial resolution of the right frontal hemosinus (Figure 5), unmasking a communication pathway for intracranial air entry and resulting in the formation of a new small intracranial air collection in the right basifrontal region measuring approximately 2.8 × 1.7 cm, consistent with delayed onset pneumocephalus (Figure 6), suggesting ongoing air entrapment and possible persistent communication with extracranial air spaces.

Following the Day 14 CT examination, the patient was managed conservatively with close neurological monitoring, supportive care, and serial clinical assessment, as there was no significant midline shift or evidence of severe mass effect at that stage. In view of the low-grade fever and fluctuating sensorium, the patient was also evaluated for possible infective complications and treated symptomatically. As the patient initially remained relatively stable without acute neurological decline, clinical follow-up was continued. 

Day 40 follow-up (critical phase): May 2025

By Day 40, the patient demonstrated marked neurological deterioration compared with prior assessments, with significantly reduced responsiveness for eight hours and features suggestive of raised intracranial pressure, including worsening sensorium and poor neurological recovery (GCS E1VTM3). The clinical picture raised concern for a secondary intracranial complication in the setting of prior traumatic injury.

**Figure 7:**
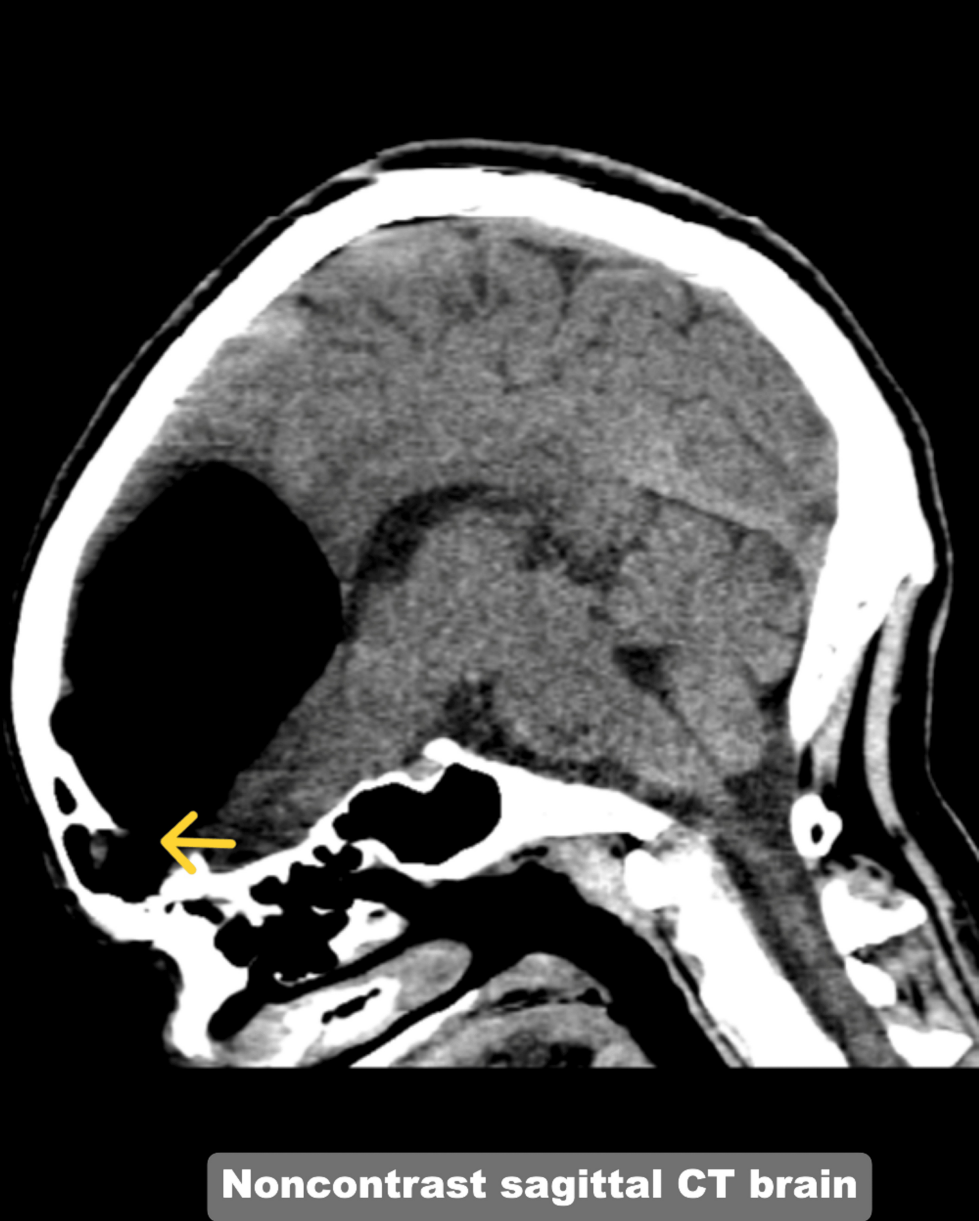
Day 40 non-contrast sagittal CT brain showing large intracerebral pneumatocele communicating with the right frontal sinus Yellow arrow demonstrates complete resolution of the right frontal hemosinus, establishing intracranial communication with resultant formation of a large intracerebral pneumatocele.

**Figure 8:**
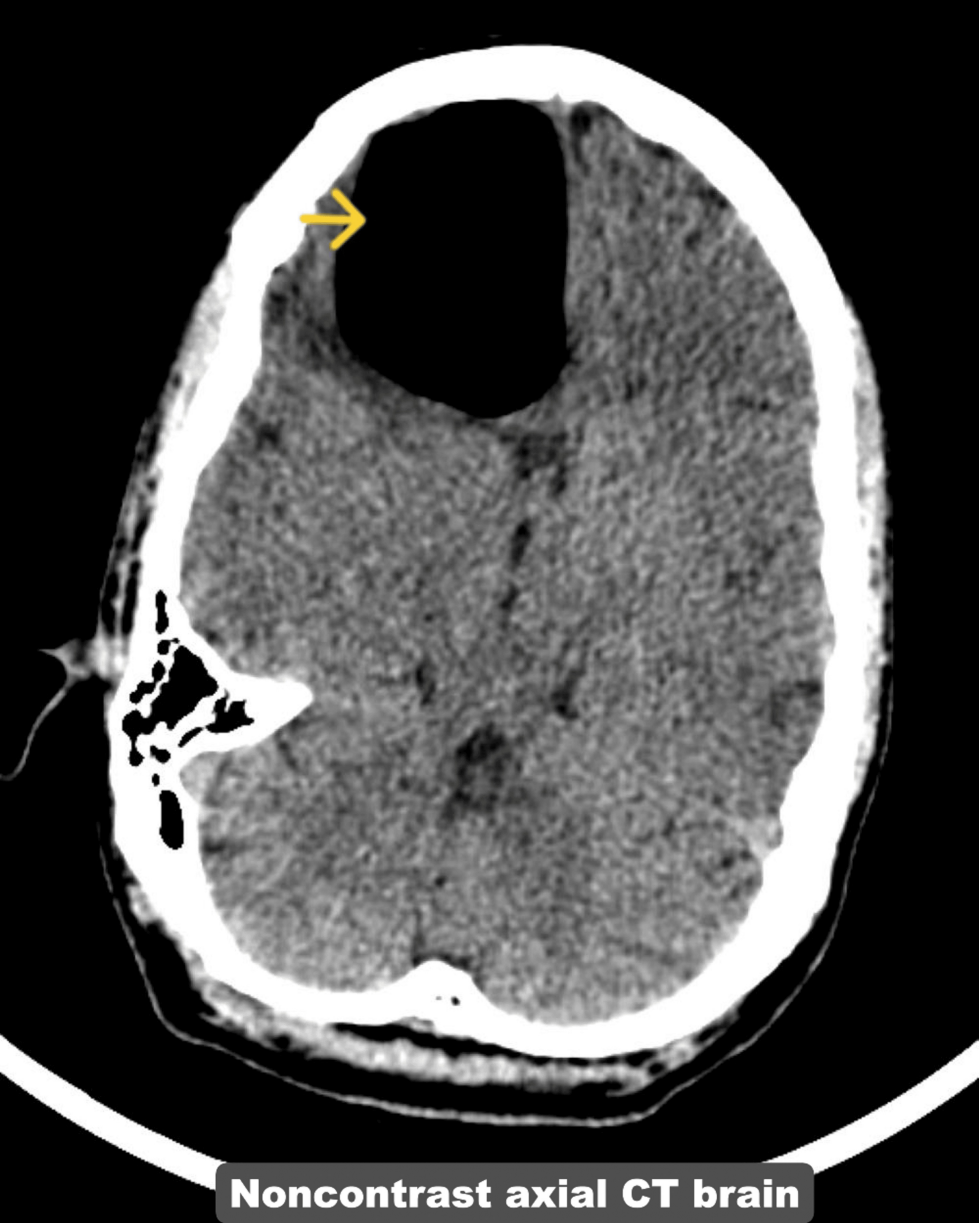
Day 40 non-contrast axial CT brain showing large intracerebral pneumatocele with mass effect Yellow arrow shows large intracerebral pneumatocele in the right basifrontal region.

Repeat non-contrast CT of the brain revealed complete resolution of the right frontal hemosinus (Figure 7), establishing a large, well-defined intracranial air collection in the right basifrontal region measuring approximately 7.9 × 4.8 cm (Figure 8). The lesion exerted significant mass effect on the adjacent brain parenchyma, evidenced by effacement of the surrounding cortical sulci and compression of the frontal horn of the lateral ventricle. There was an associated midline shift of approximately 5 mm toward the contralateral side, along with features of subfalcine herniation. The previously noted hemorrhagic contusions had largely resolved, further highlighting the progressive nature of the air collection as the primary cause of the patient’s clinical deterioration. Overall, the imaging findings were consistent with a delayed expanding pneumatocele communicating with the right frontal sinus, representing a rare but life-threatening complication of skull base trauma.

Postoperative imaging (day 41, postoperative day 0): May 2025

The patient underwent surgical intervention in the form of anterior cranial fossa base repair with fascia lata graft placement and cranioplasty for associated craniofacial defects. In the immediate postoperative period, the patient was drowsy but arousable, with early signs of neurological stabilization and no evidence of acute deterioration (GCS E3VTM4).

**Figure 9:**
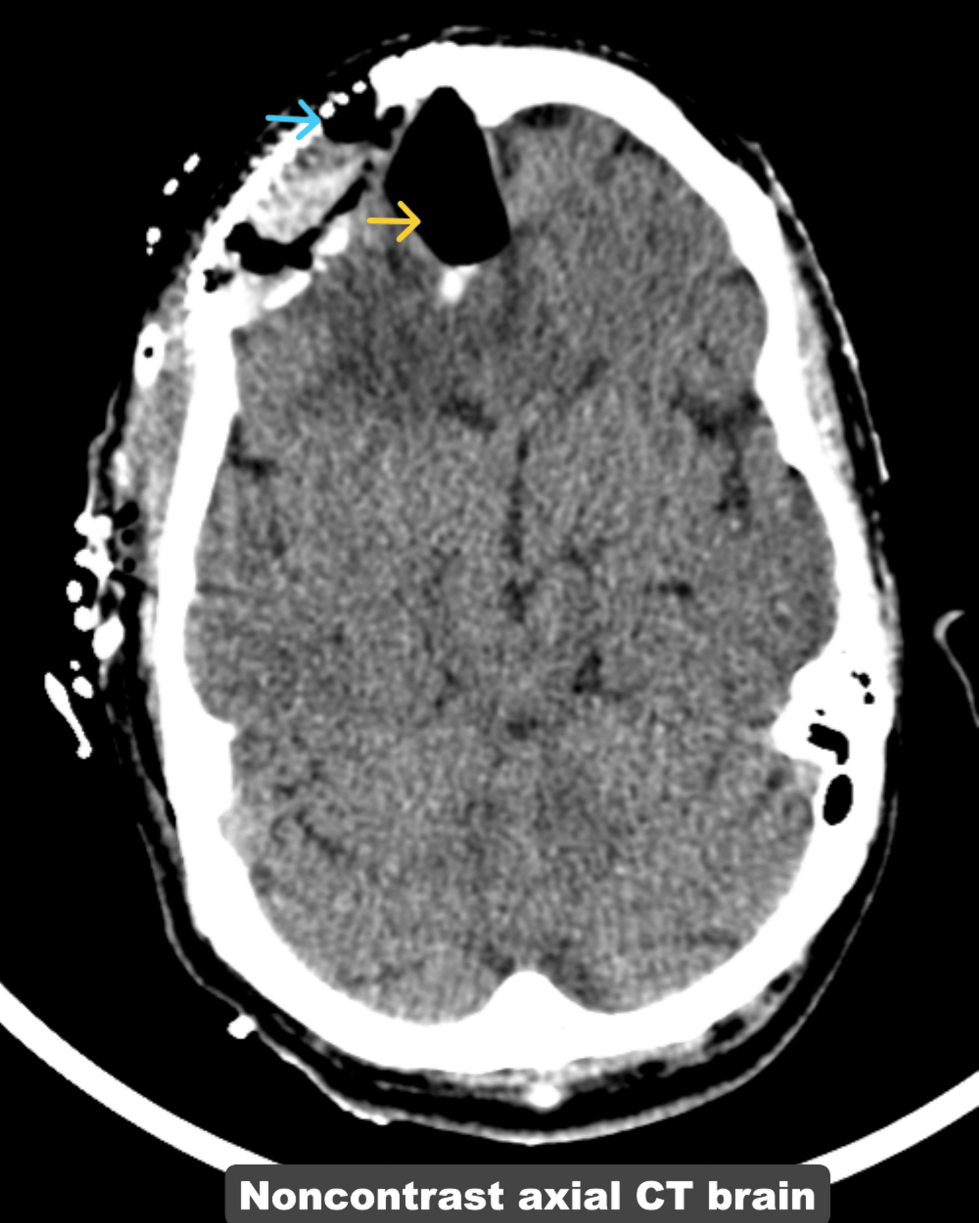
Day 41 postoperative non-contrast axial CT brain showing reduction in the pneumatocele and midline shift The yellow arrow shows a significant reduction in the previously noted intracranial air collection (intracerebral pneumatocele) in the right basifrontal region. The blue arrow shows postoperative changes in the right frontal calvaria.

Postoperative non-contrast CT of the brain (Figure 9) demonstrated a significant reduction in the previously noted intracranial air collection (intracerebral pneumatocele) in the right basifrontal region. There was a corresponding decrease in mass effect, with partial re-expansion of the adjacent brain parenchyma and reduction in midline shift compared to the preoperative study. Minimal postoperative hemorrhage was observed in the right frontal region, consistent with an expected postoperative course following decompression and repair.

On subsequent follow-up, the patient demonstrated gradual and sustained clinical improvement, with progressive recovery in GCS scores over a period of three days. Ventilatory support was successfully weaned over time, and the patient’s neurological status continued to improve without further episodes of deterioration. Overall, the postoperative course was favorable, reflecting effective surgical management of the underlying skull base defect and associated intracranial air collection.

## Discussion

Pneumocephalus is a relatively common finding following craniofacial trauma, reported in approximately 3.7%-9.7% of head injury cases, particularly when skull base fractures involve the paranasal sinuses or mastoid air cells [2]. Although most cases are small and self-limiting, the clinical importance lies in identifying those that progress to tension pneumocephalus, which can result in rapid neurological deterioration and requires urgent intervention [5,10].

The pathophysiology of pneumocephalus is primarily explained by two mechanisms. The ball-valve mechanism allows air to enter the intracranial cavity through a dural defect during episodes of increased extracranial pressure, such as coughing, sneezing, or positive-pressure ventilation, while preventing its egress, resulting in progressive accumulation; whereas, the inverted bottle mechanism involves continuous CSF leakage, which creates negative intracranial pressure and facilitates passive entry of air into the cranial cavity [4,6]. These mechanisms are particularly relevant in complex skull base fractures, where persistent communication between intracranial and extracranial compartments exists.

Intracerebral pneumatocele is a relatively rare and less well-described variant, which is defined as a localized collection of air within the brain parenchyma and behaves as a space-occupying lesion [7,9]. These lesions may progressively enlarge due to persistent air entry through a one-way mechanism and impaired resorption, eventually leading to significant mass effect. Delayed enlargement has been attributed to persistent dural defects and altered intracranial pressure dynamics [4,9].

Early clinical recognition of progressive pneumocephalus can be challenging, as initial symptoms are often non-specific and may include mild headache, nausea, dizziness, or subtle behavioral changes [11]. However, the development of fluctuating or worsening sensorium, persistent headache despite improving hemorrhagic findings, low-grade fever, or evidence of CSF rhinorrhea or otorrhea should raise suspicion for ongoing intracranial pathology [6]. Importantly, clinical deterioration following an initial period of improvement is a key warning sign of delayed pneumocephalus or intracerebral pneumatocele [10]. As intracranial air increases, features of raised intracranial pressure may develop, including vomiting, altered consciousness, and, in severe cases, signs of impending herniation [12].

CT remains the gold standard for diagnosis due to its high sensitivity in detecting even minimal intracranial air [4]. Early imaging typically demonstrates small air foci in subdural, subarachnoid, or intraparenchymal locations, often adjacent to fracture sites [4]. While these findings are frequently benign, persistence or reappearance of air on follow-up imaging is an important indicator of ongoing communication and risk of progression. Progressive radiological changes include an increase in the size of air collections and localization near skull base defects, suggesting continued air entry [9].

Critical imaging findings indicating worsening pneumocephalus include development of mass effect with effacement of cortical sulci, compression of the ventricular system, and midline shift. In advanced cases, subfalcine or transtentorial herniation may be observed. The classical “Mount Fuji sign,” characterized by separation and compression of the frontal lobes due to subdural air accumulation, represents a hallmark of tension pneumocephalus, although it is not invariably present [12]. In contrast, air within the brain tissue, forming a localized collection, represents an intracerebral pneumatocele and is distinct from extra-axial air accumulation [9].

This case illustrates a rare and clinically significant progression of a delayed, expanding intracerebral pneumatocele, a phenomenon that has been described only in limited reports in the literature. While pneumocephalus is commonly encountered following craniofacial trauma, the majority of cases are self-limiting and resolve spontaneously. Conversely, a delayed intracerebral pneumatocele with resultant mass effect represents an uncommon and potentially life-threatening complication that requires a high index of suspicion and close radiological surveillance. In the present case, the presence of extensive skull base fractures involving the frontal sinus and ethmoid air cells likely provided a persistent conduit for air entry, predisposing to delayed and progressive accumulation once the hemosinus had revolved. The anterior cranial fossa defect was identifiable on the initial imaging studies; however, it was relatively subtle in the acute setting due to the presence of extensive skull base fractures, adjacent hemorrhagic contusions, a hemosinus, and small-volume intracranial air. The right frontal hemosinus likely initially tamponaded the defect, limiting significant intracranial air entry. Following resolution of the hemosinus on subsequent follow-up imaging, a persistent communication with the frontal sinus became functionally unmasked, providing a pathway for progressive intracranial air accumulation and delayed development of the pneumatocele in the right basifrontal region. As the volume of intracerebral air increased, it began to exert a significant mass effect on the adjacent brain parenchyma, culminating in ventricular compression, midline shift, and subfalcine herniation. This stage constituted a neurosurgical emergency necessitating prompt surgical intervention for decompression and repair of dural defects for definitive management.

An important observation highlighted by this case is that initial radiological resolution of pneumocephalus does not exclude the possibility of delayed recurrence or progression. The early disappearance of intracranial air foci may create a false sense of clinical reassurance; however, persistent dural defects or occult communication with paranasal sinuses can result in reaccumulation of air over time. This underscores the importance of continued clinical vigilance and serial imaging, particularly in patients with complex skull base injuries. Early identification of warning signs, both clinical and radiological, can facilitate timely intervention and significantly improve patient outcomes.

## Conclusions

Delayed expanding intracerebral pneumatocele is a rare but clinically significant complication of skull base trauma that may manifest after an initial period of apparent improvement. It behaves as a space-occupying lesion with significant mass effect. This case underscores the importance of maintaining a high index of suspicion in patients with complex craniofacial fractures, particularly when there is discordance between clinical status and early imaging findings. Serial imaging plays a crucial role in detecting delayed progression, while vigilant clinical monitoring is essential for identifying early signs of neurological deterioration. Prompt recognition of evolving intracerebral pneumatocele, along with timely surgical intervention, is critical in preventing life-threatening complications and can significantly improve patient outcomes.
